# Comparison of sexual function in infertile women with polycystic ovary syndrome and endometriosis: A cross-sectional study

**DOI:** 10.18502/ijrm.v20i9.12066

**Published:** 2022-10-10

**Authors:** Zahra Daneshfar, Shahideh Jahanian Sadatmahalleh, Nadia Jahangiri

**Affiliations:** ^1^Department of Midwifery and Reproductive Health, Faculty of Medical Sciences, Tarbiat Modares University, Tehran, Iran.; ^2^Department of Endocrinology and Female Infertility, Reproductive Biomedicine Research Center, Royan Institute for Reproductive Biomedicine, ACECR, Tehran, Iran.

**Keywords:** Polycystic ovarian syndrome, Endometriosis, Infertility, Sexual dysfunction.

## Abstract

**Background:**

Infertility is one of the issues affecting sexual function (SF). Infertility is also one of the complications of polycystic ovary syndrome (PCOS) and endometriosis.

**Objective:**

This research seeks to assess and compare SF and the prevalence of sexual dysfunction in infertile women with PCOS and endometriosis.

**Materials and Methods:**

A cross-sectional study was carried out with a sample of 630 (210 infertile women with endometriosis, 210 infertile women with PCOS, and 210 healthy women of childbearing age as the control group). SF was assessed by the female sexual function index (FSFI). Descriptive statistics and inferential statistics were used to analyze the data. The primary outcome measured was FSFI score. Secondary outcome was hospital anxiety and depression scale score.

**Results:**

The results showed that the mean score of the total FSFI in the 2 groups of PCOS and endometriosis was lower than the control group (p < 0.001). In addition, women with higher education (university education) had a higher total FSFI.

**Conclusion:**

Sexual dysfunction rates are high in infertile women with endometriosis and PCOS. Infertility service providers in infertility centers need to pay attention to this issue.

## 1. Introduction

Worldwide estimation of infertility prevalence was 9% and had similar rates in developed and less developed countries (1). The world health organization defines infertility as “a disease of the reproductive system defined by the failure to achieve a clinical pregnancy after 12 months or more of regular unprotected sexual intercourse” (2). A person with infertility faces complex biological, psychological, social, and ethical problems (3). Infertility is a 2-person problem, and it affects the marital and sexual life of any couple (4). Sexual problems are common in infertile couples and have been reported affecting between 5% and 55% of infertile couples (5). One of the factors affecting infertile women is sexual function (SF), a key factor in physical and marital health and can significantly reduce the quality of life (6).

Endometriosis and polycystic ovary syndrome (PCOS) affect millions of women in the world (7). PCOS is the most common endocrine disorder in women of reproductive age. Sometimes this syndrome increases anxiety and tension that lead to depression, eating disorders, sexual dysfunction, and so on (8). Endometriosis is a chronic condition affecting women of reproductive age. It is induced by endometrial-like tissue outside the uterus, which induces a local inflammatory response (9). 32% of participants with endometriosis express sexual problems with intercourse pain, reduced intercourse frequency per month, and feelings of guilt against the partner (10).

In various biological windows in endometriosis and PCOS, several oxidative stress markers have been studied. It have been shown that imbalance in the levels of free radicals and disruptive antioxidants in cellular homeostasis, is supported by higher oxidants and might to reproductive and metabolic complications (7). Multiple studies have shown that oxidative stress also plays an important role in the pathophysiological mechanism of male sexual dysfunction (11-13). Based on this fact, it is assumed that oxidative stress may also play a role in the mechanism of female sexual dysfunction (14).

Considering the increasing prevalence of PCOS (from 5-10% revealed by the majority of the studies) (15) and endometriosis (estimated prevalence of 10%) (16) and their impact on fertility and sexual life, this study aims to compare the SF of infertile women having either of these diseases with the control group.

## 2. Materials and Methods 

### Participants and study design

This cross-sectional study was carried out with 630 participants selected through convenience sampling method (210 infertile women with endometriosis, 210 infertile women with PCOS, and 210 healthy women of reproductive age as the control group). Then, using the appropriate formula with α set at 0.05 and 1-β at 0.95, a sample size of 195 women was found to be required for each group. The treatment was applied to infertile women with PCOS and endometriosis who were referred to the Arash hospital clinic at Tehran in Iran, from October 2018 to December 2019. The diagnosis of PCOS was according to the Rotterdam criteria (17), and endometriosis diagnosis was confirmed using laparoscopy. Based on laparoscopic findings, we excluded women with abnormalities other than endometriosis. The subjects included 210 infertile women with a laparoscopic and/or histological diagnosis of endometriosis. Women from the control group were selected from patients referred to the Arash hospital clinic without a history of reproductive problems and diseases.

### Eligibility criteria

The inclusion criteria were the absence of any other physical diseases (such as heart and kidney diseases, diabetes, high blood pressure, chronic headaches) according to participants' medical records. The control group's inclusion criteria consisted of having at least one alive and healthy child and not being postpartum. Women who did not complete the questionnaires or met the inclusion criteria but refused to continue participation in the study were excluded from the study.

### Measures

Participants' demographic and fertility information, including age, education, occupation, body mass index (BMI), and type of infertility, were collected. The female sexual function index (FSFI) is a 19-item patient-reported outcome measure consisting of 6 separate domains of SF: desire (items 1-2), arousal (3-6), lubrication (7-10), orgasm (11-13), satisfaction (14-16), and pain (17-19) (18). Initial validation showed a good internal consistency between all scales in the general population study sample and in subgroups of FSD (female sexual dysfunction) participants and control subjects (Cranach's a = 0.82-0.97).

Test-retest reliability was acceptable (r = 0.79-0.86) (19). The Persian version of the FSFI was validated in Iran, which was a reliable and valid tool for women's sexual performance evaluation. The total score was obtained by summing the scores of 6 domains. Cut-off points for the total scale and subscales were as follows: full scale 28, desire 3.3, arousal 3.4, lubrication 3.4, orgasm 3.4, satisfaction 3.8 and pain 3.8. Scores greater than the cut-off points indicated good functions (18-20).

### Depression and anxiety 

The hospital anxiety and depression scale is designed to measure mood swings, especially anxiety and depression. On this scale, there are 7 questions about anxiety symptoms (questions 1, 4, 5, 8, 9, 12), and there are 7 questions about the symptoms of depression (questions 2, 3, 6, 7, 10, 11, 14). It is graded based on a 4-point scale (0 = never, 1 = seldom, 2 = sometimes, and 3 = always). Finally, out of 21 points recorded, scores > 8 in each category were associated with anxiety and depression. Its validity and reliability in Iran showed acceptable results (21).

### Ethical considerations

This research was approved by the Ethics Committee of Tarbiat Modares University, Tehran, Iran (Code: IR.MODARES.REC.1397.211). All procedures were in accordance with the ethical standards of the regional research committee and with the Declaration of Helsinki 1964 and its later amendments. After explaining the study's purposes, written consent and verbal assent were collected from all participants. Women were informed that their participation was voluntary, confidential, and anonymous and they were apprised of their right to withdraw from the research.

### Statistical analysis 

The collected data were analyzed through descriptive and analytic statistics using Statistical Package for Social Sciences (Statistical package for social science version 20 from American company IBM) software used to describe the data. The Levin test was used to describe the homogeneity of variances of the groups. Also, the mean of the 3 groups in terms of different variables was compared using one-way ANOVA (Analysis of variance), *t* test and Chi-square test. A p-value of < 0.05 was considered statistically significant.

## 3. Results

The results of the present study were quantitatively measured and data collection was performed using valid questionnaires.

630 subjects, including 210 women with PCOS, 210 women with endometriosis, and 210 women in the control group, were examined. In the endometriosis group, 115 (54.8%) were at stage 4, 76 (36.2%) at stage 3, and 19 (9%) at stage 1 and 2.

Comparing the mean age of the 3 groups revealed that the mean age of the PCOS group was significantly lower than that of the endometriosis and control group (p < 0.001). Also, there was a significant difference between the groups regarding the level of education, occupation, and type of infertility (p < 0.001), but the mean BMI, and duration of infertility did not differ significantly between groups (Table I).

Based on one-way ANOVA and the Dennett test (due to the heterogeneity of variances), there were significant differences in the mean score of the total FSFI and its subscales between the 3 groups (p < 0.001). As shown in table II, the mean total score of FSFI in the endometriosis group was significantly lower than PCOS and control groups, and the mean total score of FSFI in the PCOS group was significantly lower than the control group. In the endometriosis group, the score of orgasm, pain, and satisfaction subscales were significantly lower than PCOS and control groups. There were no significant differences in desire, arousal, and lubrication subscales between endometriosis and control group.

The score of desire, arousal, and lubrication subscales in the PCOS group were significantly lower than the endometriosis and control group (p < 0.001). There were no significant differences between PCOS and control groups in the orgasm subscale (Table II).

Our results showed that there was a significant relationship between education and SF, as women with college-level education had significantly lower sexual dysfunction (p < 0.001) (Table III).

Also, a comparison of the mean score of FSFI at different stages of endometriosis showed that the mean scores of FSFI in stage 3 (mean score: 24.1) and 4 (mean score: 22.3) were lower than stage 1 (mean score: 29.1) and 2 (mean score: 26.5) (p < 0.05).

**Table 1:** Comparison of demographic variables in 3 groups


**Variables**		**PCOS group**	**Endometriosis group**	**Control group**	**P-value**
Age*		30.0 ± 5.3	31.1 ± 5.3	31.4 ± 2.7	< 0.001***
BMI*		26.6 ± 4.2	25.9 ± 3.7	25.8 ± 3.7	0.08***
Type of infertility**					
	**Primary**	136 (64.8)	178 (84.8)	< 0.001****
	**Secondary**	74 (35.2)	32 (15.2)		
Duration of infertility*		3.1 ± 2.4	3.6 ± 2.7	- 0.1****
Education**					
	**Diploma (< 12 yr)**	49 (23.3)	118 (56.2)	53 (25.23)	
	**College education (≥ 12 yr)**	161 (76.7)	92 (43.8)	157 (74.77)	< 0.001***
Occupation**					
	**Housewife**	186 (88.6)	47 (22.4)	103 (49)	
	**Employed**	24 (11.4)	163 (77.6)	107 (51)	< 0.001***
HADS*					
	**Depression**	5.1 ± 4.1	5.1 ± 4.0	4.6 ± 4.0	0.27***
	**Anxiety**	7.2 ± 4.6	6.9 ± 4.0	6.6 ± 4.0	0.33***
	**Total scores**	12.3 ± 8.1	12.1 ± 7.3	11.2 ± 7.3	0.27***
*Data presented as Mean ± SD. **Data presented as number (%). ***Analysis of variance (ANOVA) or ****t test. PCOS: Polycystic ovary syndrome, BMI: Body mass index, HADS: Hospital anxiety and depression scale					

**Table 2:** Comparison of mean total FSFI scores and its subscales in 3 groups


**Variable**	**PCOS group**	**Endometriosis group**	**Control group**	P-value*
**Desire**	3.7 ± 0.8	4.1 ± 0.96	4.3 ± 1.1	< 0.001
**Arousal**	3.9 ± 0.9	4.6 ± 1.3	4.8 ± 1.5	< 0.001
**Lubrication**	4.1 ± 1.1	4.3 ± 0.7	5 ± 0.9	< 0.001
**Orgasm**	4.5 ± 0.9	3.0 ± 1.0	4.7 ± 0.8	< 0.001
**Satisfaction**	4.7 ± 1.0	3.7 ± 1.0	5 ± 0.7	< 0.001
**Pain**	3.8 ± 1.2	3.5 ± 0.9	4.5 ± 0.9	< 0.001
**Total FSFI**	25 ± 4.5	23.44 ± 2.6	28.5 ± 2.5	< 0.001
Data presented as Mean ± SD. *Analysis of variance (ANOVA). PCOS: Polycystic ovary syndrome, FSFI: Female sexual function index, P-values are adjusted for age, type of infertility, education levels, and occupation				

**Table 3:** Association between the various demographic variables and sexual dysfunction among the groups


**Variable**		**FSFI score**	**P-value**
**Education **			
	**Diploma (< 12 yr)**	24.7 ± 3.5	
	**College education (≥ 12 yr)**	26.2 ± 4.1	< 0.001*
**Occupation**			
	**Housewife**	25.7 ± 3.4	
	**Employed**	25.7 ± 4.0	0.98**
**Type of infertility **			
	**Primary**	25.3 ± 4.5	
	**Secondary**	24.7 ± 4.6	0.42**
Data presented as Mean ± SD. *Student's *t* test, **Chi-square test. FSFI: Female sexual function index			

## 4. Discussion

Sexual dysfunction is common in infertile couples, which is likely a “side effect" of frustration with their inability to have a child (15). In our study, the mean score of total FSFI in the endometriosis group was lower than in other groups. This decrease was mainly attributed to the decrease in subscale scores of orgasm, pain, and satisfaction. The rate of sexual dysfunction in the endometriosis group reported 97.1% can be attributed to the high percentage of women at stage 3 (36.2%) and 4 (54.8%) of endometriosis. Many women with endometriosis avoid sexual intercourse because of the severity of the pain. Dyspareunia affects the frequency of women having sexual intercourse, sexual desire, arousal, and orgasm (22).

A study showed that each domain of the SF (satisfaction, desire, orgasm, and pelvic problem interference) significantly affects endometriosis participants compared to healthy women (8). Our study observed that in later stages of endometriosis, the total FSFI score was lower. It was shown in a study that women with deep infiltrating endometriosis have a SF impairment (16).

Various studies revealed that endometriosis has a negative effect on the different dimensions of SF (17-19). In our study, the mean pain score in the endometriosis group was lower than the control group and the PCOS group. The cause of sexual dysfunction in endometriosis participants can be attributed to the association between deep endometriosis infiltration and dyspareunia which reduces sexual intercourses and causes lower SF (20). It seems that when experiencing this annoying situation, women with endometriosis develop negative expectations of their sexual life, threatening their sexuality (21).

PCOS is characterized by a range of hormonal and body changes, including obesity, acne, hirsutism, hyperinsulinemia, hyperprolactinemia, insufficient gonadotropin secretion, and hyperandrogenism. These changes can affect the sexuality of women with PCOS (23). Menstrual irregularity and infertility caused by PCOS can damage SF (24). Women with PCOS are also at risk of depression and anxiety that negatively affect their SF (25). In our study, the total FSFI score and its subscales were lower in the PCOS group than in the control group, and the rate of sexual dysfunction in the PCOS group was reported as 70.5%.

In contrast, a study concluded no significant difference in SF of women with PCOS with the control group (7). In the present study, scores of desire, arousal, and lubrication subscales in the PCOS group were lower than the endometriosis and control groups. In a study, desire was the most affected domain of SF in PCOS participants, highly correlated with hirsutism which had the most impact on participants' body image (26). PCOS is an endocrine disorder. Receptors for hormones (androgens, estrogens, and progesterone) are found in the brain and also in genital tissues, suggesting that they are important both in central (desire, arousal) and peripheral SF (27).

In our study, the mean BMI was higher in the PCOS group. A review study concluded that BMI and infertility affected sexuality in women with PCOS (28). In another study, BMI level higher than normal was associated with decreased desire and satisfaction, and education was one of the factors affecting SF in women with PCOS (26). The result of a meta-analysis was in line with our study conclusion. In this meta-analysis, women with PCOS, their SF and sexual attractiveness were affected (29). The findings of our study were similar to the results of another study (30). In this study, stimulation and wetting disorders were the most common sexual dysfunction in infertile women with PCOS, and SF in women with PCOS was mostly affected by infertility. In women with PCOS, the factors affecting SF included disarranged hormone levels, especially androgens, infertility, obesity, and associated problems like metabolic syndrome, body image issues, and low self-esteem. High testosterone levels directly affected sexual motivation and desire. Obesity leads to sexual inhibition, decreased sexual desire, poor body image, and low self-esteem, which in turn affects SF (31). In our study, the mean score of the orgasm subscale in the PCOS group was not significantly different from the control group.

In examining the relationship between various socio-demographic groups regarding sexual dysfunction and variables between groups, only the level of education was significantly correlated with sexual dysfunction (Tables II, III). Compared to women who do not have a university education, women with a university education were less likely to have sexual dysfunction. Our research samples were only women of reproductive age and had higher education (65% had college-level education). On the other hand, some studies have shown that higher education can protect against sexual dysfunction (23-26). This is probably due to the search for better health behaviors associated with higher education (27).

### Limitations

Female sexual dysfunction is a multidimensional disorder. It is also affected by various factors, especially the marital satisfaction of couples. Men's SF might affect women's SF. Men's erectile dysfunction, premature, and delayed ejaculation are factors that appear to play a significant role in determining female sexual dysfunction. But we had little information about the female's partners. The large number of samples in each group was the strength of our study.

## 5. Conclusion

In the present study, sexual dysfunction in the endometriosis group was less than in the control group and the PCOS group. It was lower in the PCOS group than the control group. Most sexual dysfunction in the endometriosis group was related to orgasm, satisfaction, and pain subscales, and in the PCOS group was related to desire, arousal, and lubrication subscales. Also, in our study, women with higher education level had less sexual dysfunction. Considering the impact of endometriosis and PCOS on the SF of infertile women, attention and assistance in alleviating these participants' sexual problems during the infertility diagnosis and treatment process might improve and maintain their sexual health.

##  Conflict of Interest

The authors declare that there is no conflict of interest.
